# How Doth the Little Crocodilian: Analyzing the Influence of Environmental Viscosity on Feeding Performance of Juvenile *Alligator mississippiensis*

**DOI:** 10.3390/biology5040036

**Published:** 2016-09-30

**Authors:** James R. Kerfoot, Emily Easter, Ruth M. Elsey

**Affiliations:** 1 Department of Biology, Union University, 1050 Union University Drive, Jackson, TN 38305, USA; emily.easter@my.uu.edu; 2 Louisiana Department of Wildlife and Fisheries, Rockefeller Wildlife Refuge, 5476 Grand Chenier Hwy, Grand Chenier, LA 76043, USA; relsey@wlf.la.gov

**Keywords:** feeding kinematics, habitat variation, prey capture

## Abstract

Wetland habitats are used as nursery sites for hatchling and juvenile alligators (*Alligator mississippiensis*), where they utilize prey from aquatic and terrestrial settings. However, little is known about how viscosity of the medium influences feeding performance. We hypothesized that timing and linear excursion feeding kinematic variables would be different for individuals feeding on prey above the water compared with the same individuals feeding underwater. Individuals were fed immobile fish prey and feeding events were recorded using a high speed video camera. Feeding performance was summarized by analyzing three feeding kinematic variables (maximum gape, maximum gape velocity, duration of feeding bout) and success of strike. Results of a series of paired *t*-tests indicated no significant difference in kinematic variables between feeding events above water compared with underwater. Similarity in feeding performance could indicate that prey-capture is not altered by environmental viscosity or that feeding behavior can mitigate its influence. Behavioral differences were observed during feeding events with alligators approaching underwater prey having their mouths partially opened versus fully closed when feeding above water. This behavior could be an indication of a strategy used to overcome water viscosity.

## 1. Introduction

A variety of wetland habitats are utilized by American alligators (*Alligator mississippiensis*) as nesting and nursery sites, ranging from fully aquatic to predominantly terrestrial areas [1,2]. In order to survive, hatchlings and juveniles must learn to obtain prey from both aquatic and terrestrial mediums [3]. Alligators are opportunistic predators with diets of hatchlings and juveniles being composed of insects and small fish, and as they grow into adults, shifting to incorporate larger mammals, reptiles, and birds [4,5,6,7,8,9].

Water is 700 times denser than air and presents distinct challenges to an animal attempting to accelerate body parts through the environment [10]. In previous feeding kinematic studies comparing terrestrial and aquatic feedings, Eastern box turtles (*Terrapene carolina*) and tiger salamanders (*Ambystoma tigrinum*) were able to control the amount of hyoid depression utilized in relation to the medium in which the feeding occurred [11,12]. Hyoid depression aids in expanding the oral cavity and drawing in water followed by prey. In the Eastern box turtle, hyoid depression was three times greater in aquatic compared with terrestrial settings, a behavior thought to compensate for the increased viscosity of water [12]. Interestingly, the timing variables measured (time to peak gape and time to peak hyoid depression) were not significantly different between aquatic and terrestrial settings suggesting the ability of *T. carolina* to modulate the influence of the medium to maintain performance levels [12].

These studies show how viscosity of a medium affects feeding behavior for some organisms. Could environmental viscosity evoke changes in feeding behavior of alligators as well? Few studies have been done to determine what effect, if any, viscosity of the medium has on the feeding behavior of these apex predators. The objective of this study was to determine the impact of viscosity on feeding performance of juvenile American alligators. Many experiments have been performed to determine the bite force and feeding performance of the American alligator; however, most of these studies were conducted in captive, terrestrial environments [9,13,14,15,16]. Although one study has attempted to account for underwater drag in aquatic feeding [14] and another study employed an animal-borne imaging system to describe above water and underwater foraging [15], this is the first study to explore, in a controlled laboratory setting, the influence of viscosity on feeding performance in juvenile American alligators. We hypothesized that there would be differences in timing and linear excursion feeding kinematic variables such as maximum gape, maximum gape velocity, duration of feeding bout, and successful strike for individuals feeding above compared with the same individual feeding underwater due to the differences in viscosity.

## 2. Materials and Methods

### 2.1. Subjects and Housing

Seven captive-reared juvenile *Alligator mississippiensis* were randomly acquired from Rockefeller Wildlife Refuge in Grand Chenier, Louisiana and transported live to the Union University research lab in Jackson, Tennessee in August 2015. Individuals used in this study ranged from 51.0 to 59.0 cm total length. The alligators were housed in a 1136 L holding tank filled with 200 L of aged water, which was circulated using a MagDrive 250 pump. Within the holding tank, a 0.6 m^2^ platform was provided, allowing individuals space for resting out of water. Fluorescent light fixtures were set to establish a 12:12 photoperiod. Alligators were fully adjusted to laboratory conditions for a period of 2 months. Individuals were fed daily *ad libitum* Mazuri Reptile pellets.

### 2.2. Feeding Kinematics and Experimental Design

The seven individuals were randomly placed into individual 76 L experimental tanks filled with 25 L of water. Room and water temperature were maintained at a constant 20.2 °C throughout the duration of the experiment. Individuals were not fed for 24-h prior to feeding events to induce hunger and encourage peak performance. To test the hypothesis that there would be no differences in feeding kinematic variables for individuals feeding above compared with underwater, individual fish prey (golden shiners, *Notemigonus crysoleucas*) were suspended via dissection needles either 2 cm above the waterline (above water treatment) or 2 cm below the waterline (underwater treatment); and positioned centrally with regard to the short axes of the experimental tank. Each individual was recorded feeding in both treatments, randomizing the treatment order for each individual. The use of the paired-samples design, where each individual is recorded in both treatments, serves to control for the potential effects of scaling of the dependent variables with size.

Feeding events were recorded using a Casio Exilim-FX high speed video camera at a rate of 300 fps. The camera was mounted on a tripod perpendicular to the experimental tanks. A measuring grid was affixed to the back of the tanks to standardize the measurements of the feeding events. For each individual a total of six feeding events were recorded, three in each treatment, yielding a total of 42 events analyzed.

**Figure 1:**
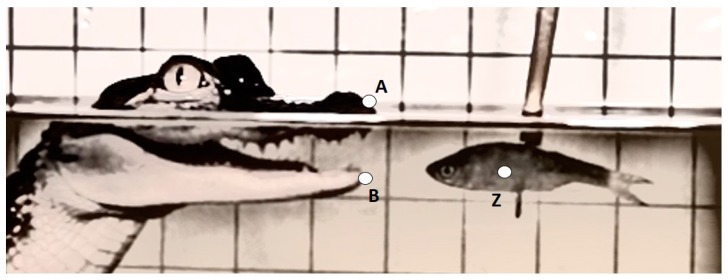
Diagram of *A. mississippiensis* portraying landmarks used to measure linear excursions during an underwater feeding event. A: anterior tip of the premaxilla; B: anterior tip of the dentary bone; Z: *N. crysoleucas* prey used in feeding event.

Three kinematic variables were measured from each recording and included: maximum gape distance (mm), maximum gape velocity (mm·s^−1^), and duration of feeding bout (s) (Table 1 and Figure 1). The linear measurement was calculated as the greatest excursion of the variable from a resting, mouth-closed position. Velocity and timing variables were measured from time zero (t_0_) which was defined as the frame prior to the mouth opening. Success of prey capture for each feeding event was also recorded. A successful prey capture was defined as an event were the alligator struck the prey, closing its mouth around it. To control for the refraction of light when traveling between mediums, and slightly distorting the size of the grid and alligator features underwater, the linear measurement for the above water treatment was calibrated to the grid above the waterline and, likewise, for the underwater treatment the measurement was calibrated to the grid below the waterline.

### 2.3. Statistical Analysis

To test the hypothesis that there would be differences in feeding kinematic variables for individuals between treatments, a series of paired *t*-tests, with a Bonferoni corrected α-level set at 0.0167, were conducted. The Bonferoni correction was used to account for three variables being measured on each individual recording (*p*-value = 0.05/3 = 0.0167). To test the hypothesis that success of prey capture was different between treatments, the success of prey capture data were analyzed using a non-parametric Wilcoxon signed ranks paired-test at an α-level of 0.050. All statistical analyses were performed using R statistical software [17].

## 3. Results

**Figure 2:**
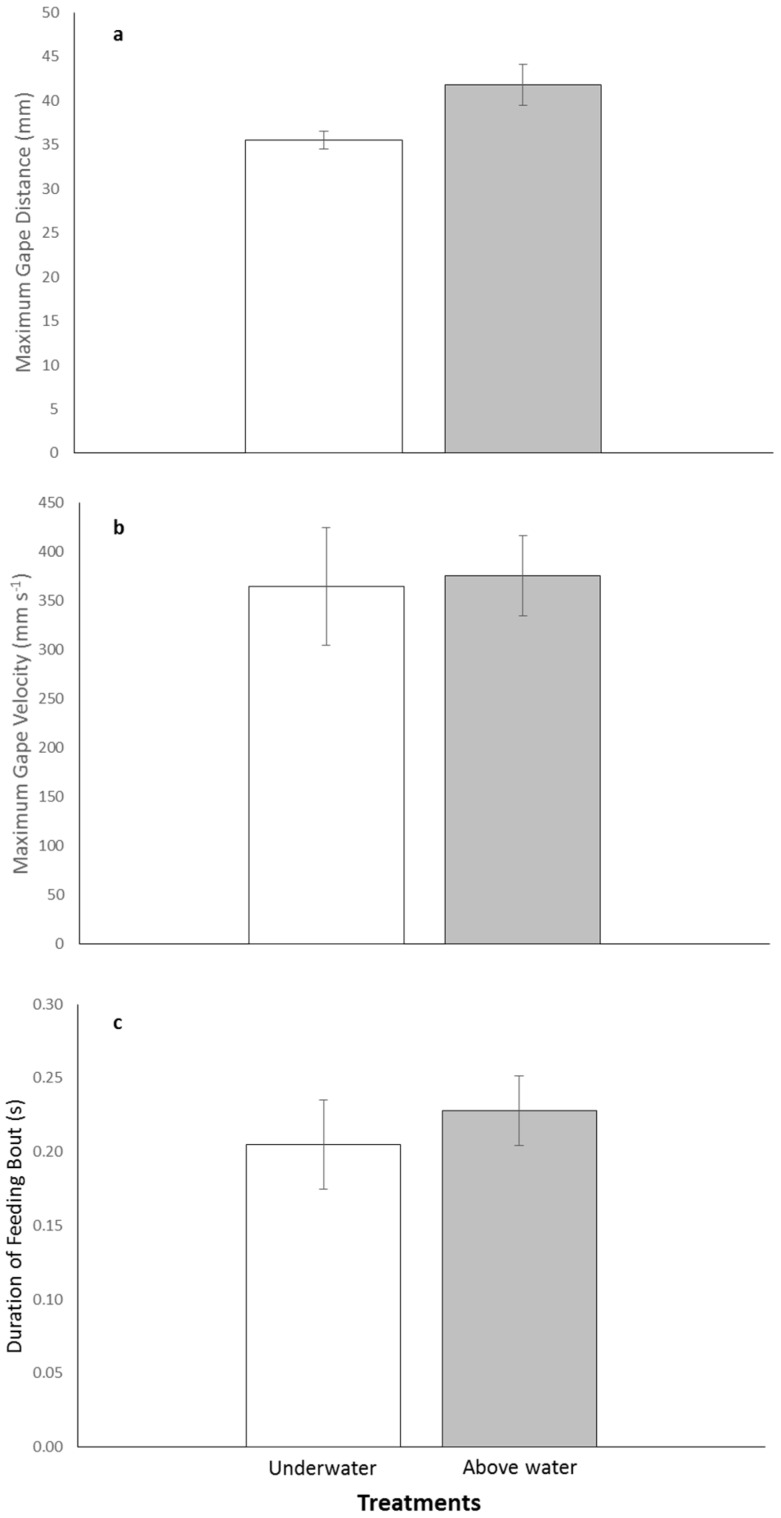
Bar graphs depicting the mean ± SE for kinematic variables: (**a**) maximum gape distance (**b**) maximum gape velocity and (**c**) duration of feeding bout. Open bars represent the underwater treatment and shaded bars represent the above water treatment.

There was variability in the kinematic parameters between treatments with individuals increasing their maximum gape distance above water compared with underwater feedings. The mean maximum gape was 41.8 ± 2.3 mm for above and 35.5 ± 1.0 mm for underwater treatments (Figure 2a). Mean maximum gape velocity was faster for the above water treatment compared with the underwater one, 375.3 ± 41.3 mm·s^−1^ and 364.2 ± 60.2 mm·s^−1^, respectively (Figure 2b). The duration of feeding both was almost identical for both treatments with the above water feeding lasting an average of 0.23 ± 0.024 s and the underwater feeding lasting 0.21 ± 0.030 s (Figure 2c). Although there was variability in the kinematic parameters between treatments, the series of paired-t tests indicated no significant differences in maximum gape distance, maximum gape velocity, and duration of feeding bout between the above and underwater feeding treatments (*p*-values > 0.0167; Table 2).

Investigating successful strikes, 81% ± 9% were successful when feeding above the water. Feeding underwater was not as successful with only a 57% ± 11% success rate. The Wilcoxon signed-ranks paired-test indicated no significant difference was detected between treatments (*p*-value = 0.242; Table 2).

## 4. Discussion

While the use of captive-raised individuals are commonly utilized in performance studies [13], concern with their use and inference to natural, wild-type counterparts has been debated [13,18,19]. Moreover, it has been shown that there are documented anatomical differences between captive and wild *A. mississippiensis* [16] that may have influences on the performance parameters measured here, warranting caution when extrapolating implications from this study to wild individuals.

Our results for the kinematic variables of above water feeding agree with a recent study [16] of kinematic scaling of the feeding mechanism for similar-aged juvenile alligators feeding on suspended strips of chicken above the water. Previous studies have indicated that viscosity of a medium affects the feeding behavior of individuals [11,12]. However, results from our study indicated that juvenile alligators feed equally well above water compared with underwater. We reject our research hypothesis, as feeding kinematic variables such as maximum gape, maximum gape velocity, duration of feeding bout, and successful strike for juvenile alligators feeding above water were comparable to the same individual feeding underwater. Similarly, maximum gape and time to maximum gape were not significantly different for *T. carolina* feeding in the two mediums [12].

Interestingly, a difference occurred where *T. carolina* was observed controlling the amount of hypoid depression used based on the medium in which it fed [12]. In the underwater setting, hyoid depression increased, not to increase suction for prey capture, but so that prey would not be pushed away by the forward motion of the predator, a process known as compensatory function. They only used enough suction to overcome the process of compensatory function. In a number of the recordings of the feeding events juvenile alligators were observed approaching prey with mouths already partially opened in the underwater treatment. This behavior, like that of *T. carolina*, would have reduced the amount of energy needed to overcome underwater viscosity, providing a compensatory function, and may explain why no difference was observed in maximum gape velocity or the duration of the feeding bout between the two treatments.

Behaviorally, individuals feeding underwater were observed several times to close their eyes during the feeding event, whereas those feeding above water did not. This behavioral difference in feeding between the mediums may signify the different senses utilized during feeding in different environmental situations. Alligators are known to have specialized sensory cells located in their integument surrounding their cranial region called integumentary sense organs (ISOs) [20,21]. Initially these ISOs were thought to aid juvenile alligators in performing an orienting response to the water surface disturbance of prey [20]. However, in alligators the highest densities are found around the teeth, inside the mouth, and at the rostral margins of the jaws, suggesting a role in discriminating food items or determining the appropriate bite force [14]. Current research suggest that these ISOs facilitate a wider array of mechanosensory abilities and conclude that ISOs function in detecting water movements, indicating when to bite based on contact of pursued prey and fine tactile discrimination of items between the jaws [21]. Interestingly, feeding above the water line permitted the juveniles to feed utilizing their visual sense and when feeding underwater they potentially utilized their ISOs (minus their vision). The duration of the feeding event and velocity of opening the jaws were similar between the mediums suggesting that even though separate senses were utilized between mediums, prey capture performance was not compromised by shifting to a different medium, but modulated to perform equally well. In a study that employed an animal-borne imaging system to investigate the foraging behavior and activity patterns of adult alligators, individuals experienced a two-fold greater success in prey-capture when attacking prey that were submerged compared with feeding on the surface [15]. In this study, prey-capture success was not significantly different; however, the prey used were immobilized, possibly influencing the feeding behavior of the predator.

A previous study investigating the scaling of prey-capture kinematics in hatchling and juvenile alligators revealed that the isometric relationship between cranial elements and body size was not transferred to the linear and timing variables of the prey-capture events [16]. The current study adds to the narrative that the kinematics of prey-capture may not be altered for this species when feeding in different mediums, at least in this size class. Kerfoot et al. [16] mentioned that studies investigating the influence of the environment on the scaling relationships of prey-capture are necessary, and while we did not directly test a scaling aspect in this study, we have laid the foundation to continue to explore the role environment plays in mitigating prey-capture in alligators. Additionally, this study documented that individuals had slightly opened mouths while underwater that possibly provided a compensatory function. It would be interesting to investigate the influence of viscosity on other anatomical structures that may provide a compensatory function related to feeding, such as the palatal valve that covers the glottis in crocodilians, preventing liquid and debris from entering in the trachea. Bite-force production has been shown to increase allometrically with head length in alligators [9], but it is not known how a change in viscosity of the medium may mitigate the bite-force produced. Future studies are required to describe the influence of viscosity on the scaling of prey-capture in alligators and its influence on force production. Correspondingly, studies investigating the role of muscle recruitment in the adductor mandibulae muscles during prey-capture event in various mediums would be beneficial.

## 5. Conclusions

Although there was variability in the kinematic parameters between treatments, the series of paired-t tests indicated no significant differences in maximum gape distance, maximum gape velocity, and duration of feeding bout between the above and underwater feeding treatments. Similarity in feeding performance of juvenile alligator between environmental mediums could indicate that prey-capture is not altered by environmental viscosity or that feeding behavior can mitigate its influence. Behavioral differences were observed during feeding events with alligators approaching underwater prey having their mouths partially opened versus fully closed when feeding above water, and this behavior could be an indication of a strategy used to overcome the influence of water viscosity.

## Figures and Tables

**Table 1:** Linear excursion, velocity, and timing variables and measurements defined when analyzing videos taken of juvenile alligators.

Variable	Units	Definition
Maximum Gape	mm	Maximum distance from the anterior tip of the premaxilla (A) to the anterior tip of the dentary bone (B). See Figure 1.
Time Zero (t_0_)		The frame where mouth begins opening.
Duration of Feeding Bout	s	Times measured from t_0_ until individual fully closes its mouth.
Maximum Gape Velocity	mm·s^−1^	Velocity measured from t_0_ until maximum gape.

**Table 2:** Results of the paired statistical analyses performed on kinematic variables. The Bonferoni corrected α-level for determining the statistical significance was set at 0.0167 for the kinematic variables and 0.050 for the variable involving successful strikes.

Variable	Statistic	Value	df	*p*-value
Maximum Gape Distance	*t*	−2.95	6	0.025
Maximum Gape Velocity	*t*	0.55	6	0.900
Duration of Feeding Bout	*t*	−0.62	6	0.560
Successful Strikes	*V*	16.50	6	0.242
